# Does COVID-19 affect sperm quality in males? the answer may be yes, but only temporarily

**DOI:** 10.1186/s12985-024-02290-5

**Published:** 2024-01-23

**Authors:** Qi-Feng Zhang, Yu-Ji Zhang, Sheng Wang, Yu Wei, Han Zhang, Feng Li, Yong-Quan Deng

**Affiliations:** 1 Department of Andrology, Guilin People’s Hospital, 541002 Guilin, China; 2 Department of Medical Record Management, The First Affiliated Hospital of Hainan Medical University, 571137 Haikou, China; 3 Department of Urology, Guilin People’s Hospital, 541002 Guilin, China

**Keywords:** COVID-19, SARS-CoV-2, Sperm quality, Semen parameters, Infertility investigations

## Abstract

**Background:**

The Corona Virus Disease 2019 (COVID-19) pandemic has raised concerns regarding its potential impact on male reproductive health. However, the impact of COVID-19 on sperm quality remains uncertain. This retrospective study aimed to investigate the short-term and relatively long-term effects of COVID-19 infection on sperm quality.

**Methods:**

A total of 85 males with fertility requirements, who underwent semen evaluation at Guilin People’s Hospital between June 2022 and July 2023, were included in the study. Changes in semen parameters were analyzed across three specific timeframes: within 6 months before COVID-19 infection, within 3 months after COVID-19 infection, and 3–6 months after COVID-19 recovery.

**Results:**

The results revealed that the sperm concentration and total sperm number were significantly lower after infection compared to before, while in the recovery period, the sperm concentration, total sperm count, progressive motility, and normal morphology significantly increased. Comparing the three periods, the most significant difference was observed in sperm concentration, which exhibited a significant decrease after infection but returned to normal levels after recovery from COVID-19.

**Conclusions:**

These findings suggest that COVID-19 may exert some impact on sperm quality, particularly evidenced by decreased sperm concentration post-infection. Fortunately, these effects on semen parameters appear to be temporary, with gradual restoration of semen parameters within 3–6 months after recovery. However, further research is needed to explore the underlying mechanisms and long-term implications of these observed changes in semen parameters.

## Introduction

Corona Virus Disease 2019 (COVID-19) is an acute infectious disease caused by severe acute respiratory syndrome coronavirus 2 (SARS-CoV-2). SARS-CoV-2 can have potential negative effects on multiple human systems and organs by binding to angiotensin-converting enzyme 2 (ACE2) receptors [1, 2]. Therefore, any tissue expressing ACE2 receptors may become a target for SARS-CoV-2 infection, and the human testicles are no exception. Research has confirmed that the human testis is a potential target of SARS-CoV-2 infection, and ACE2 was predominantly enriched in spermatogonia, Leydig cells, and Sertoli cells [3]. In patients with COVID-19 infection, spermatogenic and endocrine (Leydig cells) testicular functions decreased [4], moreover, SARS-CoV-2 can enter Human Leydig Cells through a distinct pathway and Affects Testosterone Production In Vitro [5], even affecting testicular size and elasticity [6]. Although the risk of the presence of SARS-CoV-2 in semen appears to be extremely low in the acute or convalescent stages, the impact of the virus on male semen quality is indeed significant [7, 8]. Existing evidence suggests that COVID-19 in men has a significant and long-term effect on sperm quality, especially on sperm concentration and total motility [9]. A prospective cross-sectional study reported that SARS-CoV-2 can be detected in semen in a small proportion of men who recovered from COVID-19, and around 25% of individuals who recovered from COVID-19 demonstrated oligo-crypto-azoospermia [10]. Similarly, a study compared semen samples from COVID-19-recovered males and those who had never had COVID-19, revealing lower sperm concentrations in the COVID-19 group, indicating potential adverse effects on male fertility, while sperm motility and morphology showed no significant differences between the groups [11]. A recent systematic review and meta-analysis show that even though the viral RNA is usually undetectable in semen, COVID-19 affects sperm concentration, total sperm count, and sperm volume [12]. However, some studies have shown that mild COVID-19 may have no detrimental effect on semen parameters [13], and there was no significant difference in spermiogram results between patients who presented for infertility before and during the COVID-19 pandemic [14], and even during the COVID-19 pandemic the sperm concentration, total sperm count, and total motility in male partners of infertile couples were better than those in the COVID-19 prepandemic [15]. Further research is needed to reveal the impact of COVID-19 on male sperm quality during different periods and populations of the COVID-19 pandemic.

This study aims to examine the short-term and relatively long-term effects of COVID-19 on male fertility by observing the changes in semen parameters before and after COVID-19 infection, as well as 3–6 months after COVID-19 recovery, among males with fertility requirements.

## Materials and methods

### Study procedures and population

**Fig. 1:**
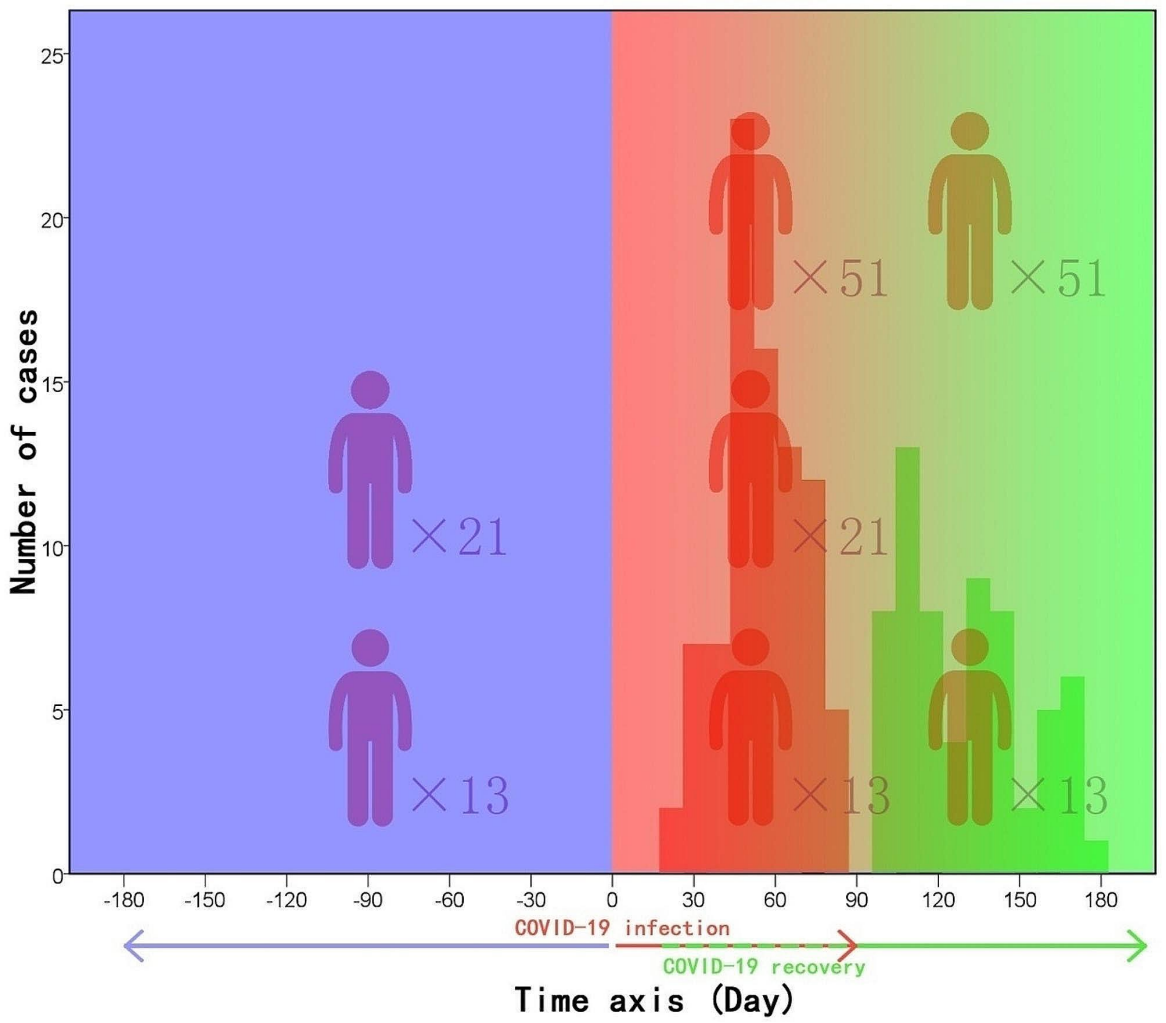
Timeline of case distribution

This is a retrospective research, with adult males who have been admitted to Guilin People’s Hospital for infertility investigations between June 2022 and July 2023 and underwent semen evaluation within 3 months after COVID-19 infection, within 6 months before COVID-19 infection, and/or 3–6 months after COVID-19 recovery, respectively. After excluding patients with male azoospermia, cryptorchidism, varicocele, genitourinary tract infection, and any urogenital operations during this period, as well as patients who have received treatments such as gonadotropin therapy, antioxidants, or herbal remedies to improve sperm quality, a total of 85 males were included in the final analysis, and all of them exhibited mild or moderate clinical symptoms, with no severe or hospitalized cases. Among these individuals, 13 patients underwent semen analysis within 3 months after COVID-19 infection, within 6 months before COVID-19 infection, and 3–6 months after COVID-19 recovery; 21 patients underwent semen analysis within 3 months after COVID-19 infection and within 6 months before COVID-19 infection; and 51 patients underwent semen analysis within 3 months after COVID-19 infection and 3–6 months after COVID-19 recovery, respectively (Fig. 1; Table 1). The study was approved by the Medical Ethics Committee of Guilin People’s Hospital.

### Covid-19 diagnosis and semen analysis

The diagnosis and recovery of COVID-19 were confirmed by clinical symptoms and positive SARS CoV-2 test (RT-PCR or rapid antigen test). Semen samples were collected in sterile containers by masturbation after 2–7 days of abstinence. The Sperm Quality Analyzer (SQA-V, Medical Electronic Systems Co., Ltd.) was used for the semen analysis within 60 min after ejaculation and liquefaction. Semen volume, PH, sperm concentration, total sperm number, progressive motility (PR), total motion (PR + NP) (NP, Non-progressive motility), and normal morphology were measured according to the guidelines outlined by the World Health Organization (WHO, 5th Edition). Sperm morphology was assessed using modified Papanicolaou staining by light microscope.

### Statistical analysis

Data analysis was performed using IBM SPSS Statistics for Windows, Version 24 (IBM Corp., Armonk, New York, United States). Compliance of variables with a normal distribution was analyzed with the Shapiro-Wilk Test and homogeneity of variance by the Levene’s Test. Continuous variables were presented as means with standard deviations (mean ± SD) or medians (25–75%). Categorical variables were represented as numbers with percentages. The Paired T-test or Wilcoxon Signed-Ranks Test was used for a comparative analysis of sperm parameters before and after COVID-19 infection in the patients, as well as a comparative analysis of sperm parameters after COVID-19 infection and COVID-19 recovery. One-way repeated Measures ANOVA or Friedman Test was used for the comparison of semen parameters between the three time periods before and after infection. All statistical tests were two-tailed, and *P* < 0.05 was considered statistically significant.

## Results

The demographic and clinical characteristics of the patients are given in Table 1.

**Table 1:** Demographic characteristics of the patients

	Before COVID-19 infection	within 3 months after COVID-19 infection	3–6 months after COVID-19 recovery
n	34	85	64
Age(years)	35.59 ± 5.41	34.02 ± 5.18	33.91 ± 6.06
BMI(kg/m^2^)	23.20 ± 1.87	23.17 ± 1.92	23.23 ± 1.92
Hypertension, n	2	7	6
Diabetes, n	0	2	2
No fever (< 37.3 °C), n (%)		0	
Low grade fever (37.3–38.0 °C), n (%)		17(20%)	
Moderate fever (38.1–39.0 °C), n (%)		48(56.5%)	
High grade fever (> 39.0 °C), n (%)		20(23.5%)	
Course of disease(day)		6.00(5.25-8.00)	
Time after infection(day)		55.24 ± 14.57	128.50(110.50–147.00)

34 patients underwent semen analysis within 3 months after COVID-19 infection and within 6 months before COVID-19 infection. The results revealed that the sperm concentration and total sperm number were significantly lower after infection compared to before (*p* = 0.023 and *p* = 0.003). However, there were no significant changes observed in PH, semen volume, total motility, progressive motility, and normal morphology of sperm (*p* = 0.812, *p* = 0.425, *p* = 0.103, *p* = 0.135, and *p* = 0.230, respectively) (Table 2).

**Table 2:** Comparison of semen parameters before and after COVID-19 infection

	before COVID-19 infection	within 3 months after COVID-19 infection	t / Z	*p*-value
n	34	34		
Sexual abstinence (day)	4.50(3.00–6.00)	5.00(3.00–6.00)	-0.049	0.961 ^a^
PH	7.17 ± 0.31	7.20(7.00-7.40)	-0.238	0.812^a^
Semen volume (mL)	3.13 ± 1.26	3.00(2.00–4.00)	0.807	0.425^b^
Sperm concentration (×10^6^/mL)	56.79 ± 29.38	44.85(28.33–69.75)	2.391	0.023^b^
Total sperm number(×10^6^ per ejaculate)	172.21 ± 93.50	138.50 ± 70.60	3.155	0.003^b^
Total motility (%)	44.06 ± 9.90	41.38 ± 12.41	1.678	0.103^b^
Progressive motility (%)	33.12 ± 11.05	30.71 ± 11.78	1.534	0.135^b^
Normal morphology (%)	5.00(4.00–9.00)	5.00(3.00–8.00)	-1.200	0.230^a^

^a^ Wilcoxon Signed-Ranks Test

^b^ Paired Samples Test

64 patients underwent semen analysis within 3 months after COVID-19 infection and 3–6 months after COVID-19 recovery. Compared with after infection, the results showed a significant increase in sperm concentration, total sperm number, progressive motility, and normal morphology during the recovery period. However, no significant changes were observed in semen pH and volume (Table 3).

**Table 3:** Comparison of semen parameters after COVID-19 infection and COVID-19 recovery

	within 3 months after COVID-19 infection	3–6 months after COVID-19 recovery	t / Z	*p*-value
n	64	64		
Sexual abstinence (day)	4.50(3.00–6.00)	4.00(3.00–5.00)	0.509	0.613^b^
PH	7.20(7.00-7.30)	7.20(7.00-7.30)	0.502	0.617^b^
Semen volume (mL)	3.00(2.00–4.00)	3.25(2.13-4.00)	-1.250	0.211^a^
Sperm concentration (×10^6^/mL)	44.85(28.03–68.75)	57.90(42.63-74.00)	-4.433	< 0.001^b^
Total sperm number(×10^6^ per ejaculate)	130.65(82.40-171.63)	170.50(111.53-239.23)	-4.356	< 0.001^b^
Total motility (%)	41.75 ± 13.10	43.11 ± 10.91	-1.241	0.219^b^
Progressive motility (%)	29.55 ± 11.55	31.78 ± 10.52	-2.566	0.013^b^
Normal morphology (%)	5.00(3.00–8.00)	6.00(4.00–8.00)	-2.303	0.021^a^

^a^ Wilcoxon Signed-Ranks Test

^b^ Paired Samples Test

**Fig. 2:**
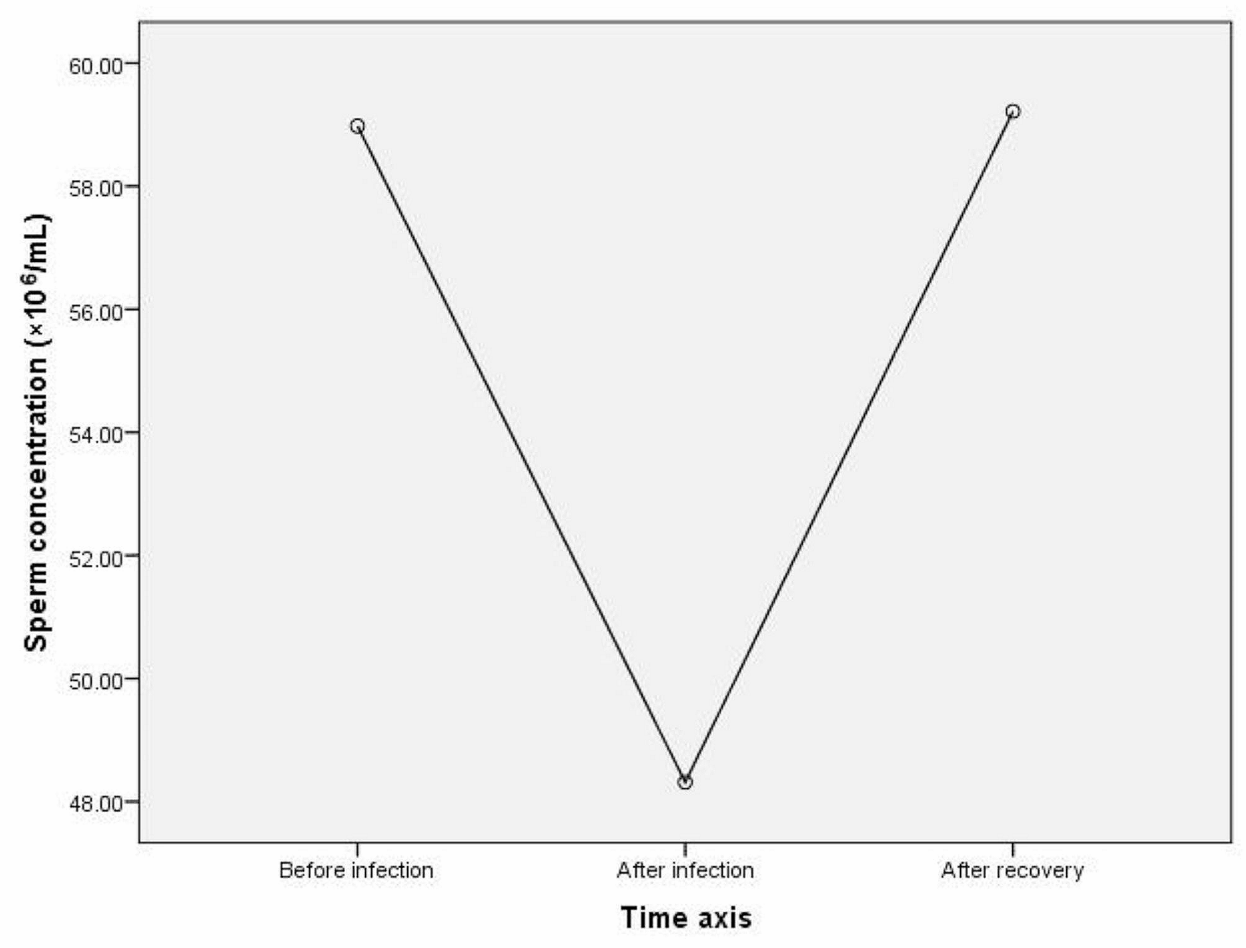
Changes in sperm concentration over three time periods: within 6 months before COVID-19 infection, within 3 months after COVID-19 infection, and during the 3–6 months after COVID-19 recovery

**Fig. 3:**
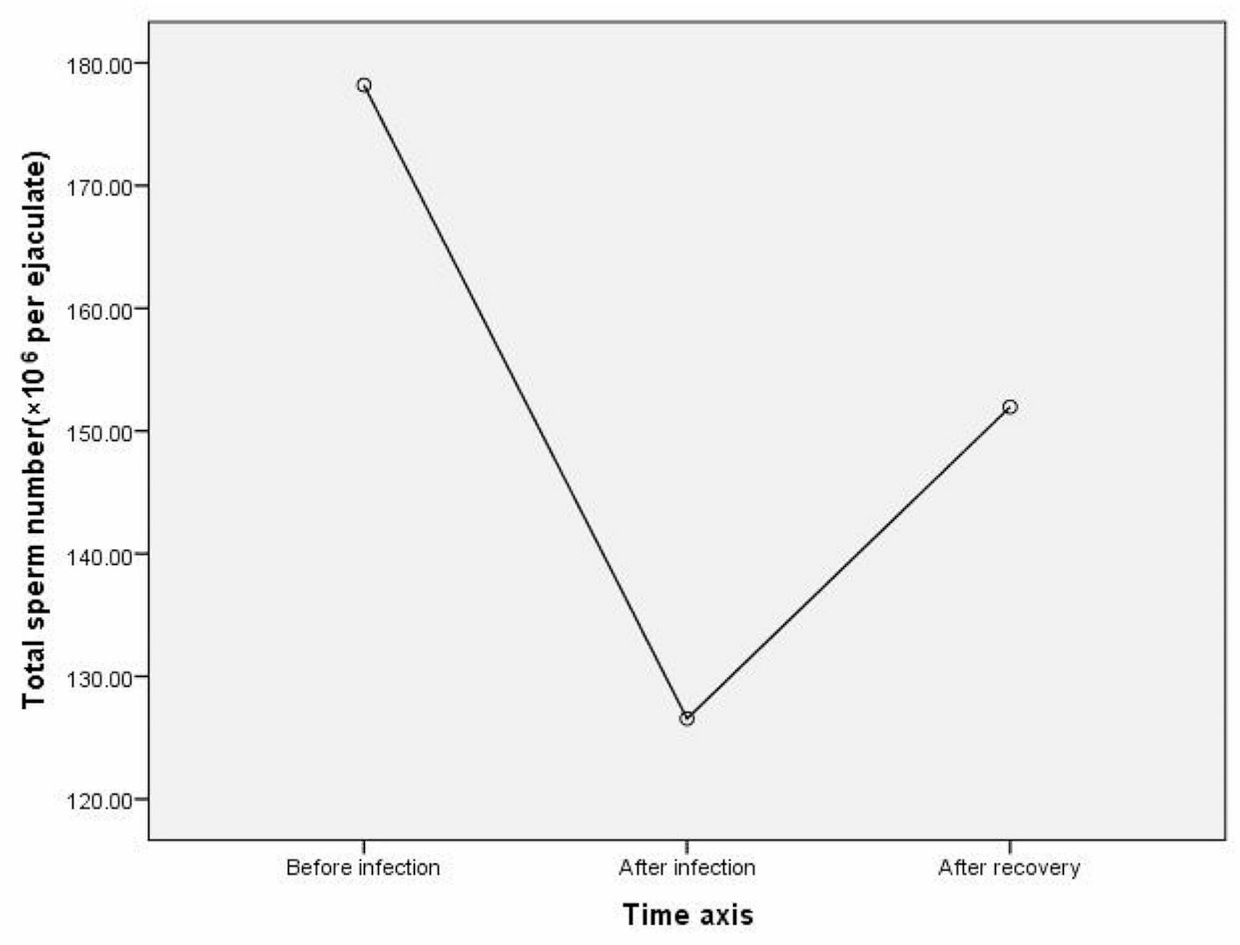
Changes in total sperm number over three time periods: within 6 months before COVID-19 infection, within 3 months after COVID-19 infection, and during the 3–6 months after COVID-19 recovery

13 patients underwent semen analysis within 3 months after COVID-19 infection, within 6 months before COVID-19 infection, and 3–6 months after COVID-19 recovery. The results of One-Way Repeated Measures ANOVA showed significant differences in sperm concentration and total sperm number among the three time periods, with F = 4.686, *p* = 0.019 and F = 3.517, *p* = 0.046, respectively. The results of LSD (least significant difference) multiple comparisons showed that the sperm concentration before infection was higher than that within 3 months after infection (*p* = 0.042), and the sperm concentration within 3–6 months after COVID-19 recovery was higher than that within 3 months after infection (*p* = 0.012). There was no significant difference in sperm concentration between the first 3 months of infection and 3–6 months after COVID-19 recovery (*p* = 0.950); The total sperm number before infection was greater than within 3 months after infection (*p* = 0.012), while there was no significant difference in the total sperm number between 3 and 6 months after COVID-19 recovery and within 3 months after infection (*p* = 0.157, *p* = 0.291) (Table 4; Fig. 2, and Fig. 3).

**Table 4:** Comparison of semen parameters before, after COVID-19 infection and after recovery frome COVID-19

	Before COVID-19 infection ①	within 3 months after COVID-19 infection ②	3–6 months after COVID-19 recovery ③	F / χ²	*p*-value	Multiple comparisons
n	13	13	13			
Sexual abstinence (day)	4.31 ± 1.49	4.31 ± 1.44	4.62 ± 1.26	0.156	0.857 ^a^	
PH	7.21 ± 0.32	7.23 ± 0.41	7.12 ± 0.40	0.673	0.519 ^a^	
Semen volume (mL)	3.31 ± 1.38	3.04 ± 1.38	3.31 ± 1.42	1.097	0.350 ^a^	
Sperm concentration (×10^6^/mL)	58.98 ± 31.87	48.32 ± 29.46	59.22 ± 35.49	4.686	0.019 ^a^	①>②*p* = 0.042 ③>② *p* = 0.012
Total sperm number(×10^6^ per ejaculate)	178.19 ± 88.55	126.55 ± 62.88	151.96 ± 89.29	3.517	0.046 ^a^	①>② *p* = 0.012
Total motility (%)	44.15 ± 9.80	39.46 ± 10.93	42.69 ± 12.80	1.816	0.199 ^a^	
Progressive motility (%)	31.15 ± 11.98	26.69 ± 10.52	30.77 ± 12.34	2.326	0.119 ^a^	
Normal morphology (%)	5.00(3.50-9.00)	5.23 ± 2.92	5.00(3.50–8.50)	2.450	0.294 ^b^	

^a^ One-Way Repeated Measures ANOVA

^b^ Friedman Test

## Discussion

The Corona Virus Disease 2019 (COVID-19) pandemic has not only been a public health crisis but has also raised concerns about its effects on male reproductive health, particularly sperm quality. Following adjustments to COVID-19 prevention and control measures in China in December 2022, there was a significant surge in infections in a very short period [16, 17], and almost all of our cases were infected in this period. Taking into account the cycle from sperm production to maturation, we collected cases of individuals who underwent semen analysis within 3 months after being infected with COVID-19 and traced them back 6 months before infection or 3–6 months after recovery. The comparison revealed a significant post-infection decrease in sperm concentration and total sperm count, as well as varying degrees of decrease in progressive motility and normal morphology, although not statistically significant. Compared to previous studies [18–20], although all have confirmed the impact of infection on sperm, we did not observe a decrease in sperm motility or normal morphology. However, our further investigation during the recovery phase demonstrated a significant increase in sperm concentration, total sperm number, progressive motility, and normal morphology compared to the post-infection period. This confirms, at least in line with previous research, that infection is indeed an important factor affecting sperm quality, as there is consistency between the decrease in sperm concentration and total sperm count after infection and the subsequent recovery in sperm concentration. Similarly, studies in patients with moderate to severe COVID-19 patients have also shown that the negative impact of SARS-CoV-2 on testicular function dissipates within 3 months [21]. Our subsequent research across three specific time frames, before COVID-19 infection, within 3 months after infection, and 3–6 months after recovery, reaffirmed a noteworthy decrease in sperm concentration and total sperm count after infection, but within 3 to 6 months after recovery, the sperm concentration returned to the pre-infection level. This provides conclusive evidence for the impact of COVID-19 on sperm cycle-dependent parameters, with a possible mechanism involving temporary suppression of sperm production due to SARS-CoV-2 infection, specifically through the temporal immune-mediated arrest of active meiosis. However, this mechanism is temporary [22].

Although our study focused on investigating the impact of COVID-19 on sperm quality, understanding the specific mechanisms involved would provide valuable insights into the pathophysiology of COVID-19-related reproductive dysfunction. SARS-CoV-2 viral RNA was not detected in semen samples from the majority of COVID-19 patients, but SARS-CoV-2 may interfere with the reproductive system through synergistic mechanisms such as fever, Inflammation or oxidative stress, rather than direct viral infection [23]. Fever, a common symptom of COVID-19, may affect the sperm quality of infected men similarly to other febrile diseases, due to increased testicular temperature and disruption in the thermoregulatory systems. Fever caused by SARS-CoV-2 infection can have varying degrees of negative effects on sperm parameters, and SARS-CoV-2 can have long-term adverse effects on testicles through immune or inflammatory reactions after fully recovered patients [24]. A meta-analysis also confirmed that COVID-19-induced fever significantly reduced sperm concentration and progressive sperm motility [9]. However, the current research results do not seem to be consistent as to whether fever is the main factor affecting the quality of sperm in infected men. Some studies have found that fever may only be related to semen concentration [25], and even some studies have shown that the severity of COVID-19 infection and the presence of fever were not correlated with sperm characteristics [7].

Oxidative stress and cell damage are also important mechanisms by which COVID-19 may affect testicular function. Moghimi et al. found that the formation of reactive oxygen species (ROS) in the testes of COVID-19 patients increased, while glutathione disulfide (GSH) was inhibited, which confirmed that COVID-19 infection may lead to the interruption of Spermatogenesis through oxidative stress pathway and subsequently induces apoptosis [26]. Another study also suggests that SARS-CoV-2 infection can induce oxidative stress reaction, leading to increased levels of intracellular oxidative stress and DNA damage, which has a negative impact on spermatogenesis. Supplementation of antioxidants, such as N-acetylcysteine (NAC), can indeed improve sperm concentration and acrosome reaction while reducing ROS and oxidative damage to sperm DNA [27]. In addition, testicular injury associated with SARS-CoV-2 infection may be an indirect consequence of exposure to systemic inflammation and/or SARS-CoV-2 antigens [28], and the concentration of leukocytes in the semen of patients with COVID-19 is increased, and the immune factors, including interleukin-6 (IL-6), tumor necrosis factor-α (TNF-α) and monocyte chemoattractant protein-1 (MCP-1) is increased, which may affect spermatogenesis and interfere with fertility due to testicular immune response [29]. Furthermore, the side effects of medications used to treat or alleviate symptoms associated with COVID-19 infection are also a significant consideration. However, some studies have indicated that these drugs, such as favipiravir and hydroxychloroquine, do not exert a significant impact on sperm quality [30, 31].

This study observed the dynamic changes in semen parameters over relatively extended periods before, during, and after COVID-19 infection within the same cohort, eliminating potential individual differences in results to more accurately reflect the short-term and long-term impacts of COVID-19 on sperm quality, providing a more comprehensive dataset for a deeper understanding of its effects on male reproductive health. The overwhelming majority of our cases were infected with the Omicron variant. While the collected cases exhibit symptoms of fever, it cannot be confirmed whether fever is the primary factor leading to impaired sperm quality in infected males, but it doesn’t seem to be too much of a concern. The mechanisms through which COVID-19 causes a decline in sperm quality may be the result of multiple factors such as fever, inflammation, immune response, or oxidative stress. Fortunately, these mechanisms causing sperm damage are likely to be temporary, reversible, and seemingly self-healing. However, due to limitations in the research methodology and patient data source, our study only focused on patients with mild to moderate cases of COVID-19 and did not include severe cases. Furthermore, most COVID-19 patients have received treatment with Nonsteroidal Antiinflammatory Drugs (NSAIDs) and/or a variety of Chinese Patent Medicines to alleviate symptoms. However, the potential impact of these medications on sperm quality cannot be ruled out. Additionally, due to adjustments in local epidemic prevention policies and the evolving epidemiology of COVID-19, the vast majority of the population has been infected with COVID-19 within a relatively short timeframe, which limits the number of participants we can collect. A smaller sample size and potential selection bias may have an adverse impact on the conclusions.

## Conclusion

In conclusion, this study suggests that COVID-19 may indeed have an impact on sperm quality, as evidenced by the significant reduction in sperm concentration and total sperm number after infection. However, within 3–6 months after the recovery of COVID-19, almost all of the decreased semen parameters recovered, even most of them reaching the pre-infection level. Therefore, we believe that COVID-19 may have an impact on sperm quality, but only temporarily. However, further research is needed to confirm this hypothesis and elucidate the potential mechanisms and long-term effects of COVID-19 on male fertility, to provide more accurate reproductive advice and management strategies for patients with COVID-19.

## Data Availability

The datasets used and/or analyzed during the current study are available from the corresponding author upon reasonable request.

## Abbreviations

COVID-19: Corona virus disease 2019
SARS-CoV-2: Severe acute respiratory syndrome coronavirus 2
ACE2: Angiotensin converting enzyme 2
ROS: Reactive oxygen species
